# Spectroscopic Studies of the Super Relaxed State of Skeletal Muscle

**DOI:** 10.1371/journal.pone.0160100

**Published:** 2016-08-01

**Authors:** Leonardo Nogara, Nariman Naber, Edward Pate, Marcella Canton, Carlo Reggiani, Roger Cooke

**Affiliations:** 1 Dipartimento di Scienze Biomediche, University of Padua, Padua Italy; 2 Department of Biochemistry/Biophysics, University of California San Francisco, San Francisco, California, United States of America; 3 Voiland School of Bioengineering, Washington State University, Pullman, Washington, United States of America; University of Minnesota Twin Cities, UNITED STATES

## Abstract

In the super-relaxed state of myosin, ATPase activity is strongly inhibited by binding of the myosin heads to the core of the thick filament in a structure known as the interacting-heads motif. In the disordered relaxed state myosin heads are not bound to the core of the thick filament and have an ATPase rate that is 10 fold greater. In the interacting-heads motif the two regulatory light chains appear to bind to each other. We have made single cysteine mutants of the regulatory light chain, placed both paramagnetic and fluorescent probes on them, and exchanged them into skinned skeletal muscle fibers. Many of the labeled light chains tended to disrupt the stability of the super-relaxed state, and showed spectral changes in the transition from the disordered relaxed state to the super-relaxed state. These data support the putative interface between the two regulatory light chains identified by cryo electron microscopy and show that both the divalent cation bound to the regulatory light chain and the N-terminus of the regulatory light chain play a role in the stability of the super-relaxed state. One probe showed a shift to shorter wavelengths in the super-relaxed state such that a ratio of intensities at 440nm to that at 520nm provided a measure of the population of the super-relaxed state amenable for high throughput screens for finding potential pharmaceuticals. The results provide a proof of concept that small molecules that bind to this region can destabilize the super-relaxed state and provide a method to search for small molecules that do so leading to a potentially effective treatment for Type 2 diabetes and obesity.

## Introduction

In resting skeletal muscle myosin is found in two states, one in which it is bound to the core of the thick filament with a highly inhibited ATPase activity, and one in which it is disordered, free to diffuse in the inter-filament space and has a higher ATPase activity. Ferenczi et al [1] over 35 years ago, showed the existence of an inhibited state of myosin in living relaxed fibers via a comparison between the ATPase activity of purified myosin from frog muscle, and the much lower metabolic rate of living fibers. A similar conclusion was reached for rabbit muscle myosin [2]. However, the inhibited state was not observed in *in vitro* muscle assays for many years, in spite of numerous attempts. The inhibited state was finally monitored by measuring single cycle ATP turnovers initiated by incubation of skinned fibers in a fluorescent analog of ATP, followed by chase with unlabeled ATP [3]. The inhibited state was called the Super Relaxed State (SRX). Although the presence of an ordered state of myosin heads in the relaxed fibers had been known for decades [4–7], the role of this state in the kinetics of the myosin ATPase in vertebrate striated muscle was not appreciated. Correlations between the stability of the SRX, measured kinetically, and the stability of the array of myosin heads bound to the core of the thick filament as measured by x-ray diffraction or by EM, has suggested the hypothesis that the inhibited state is associated with an ordered structural state [3, 8]. Testing this hypothesis is one of the goals of the present work. Myosin heads in the SRX are in equilibrium with myosin heads that are disordered and moving freely in the inter-filament space, which we term the disordered relaxed state (DRX). These myosin heads have the faster ATPase activity observed for purified myosin.

**Fig 1:**
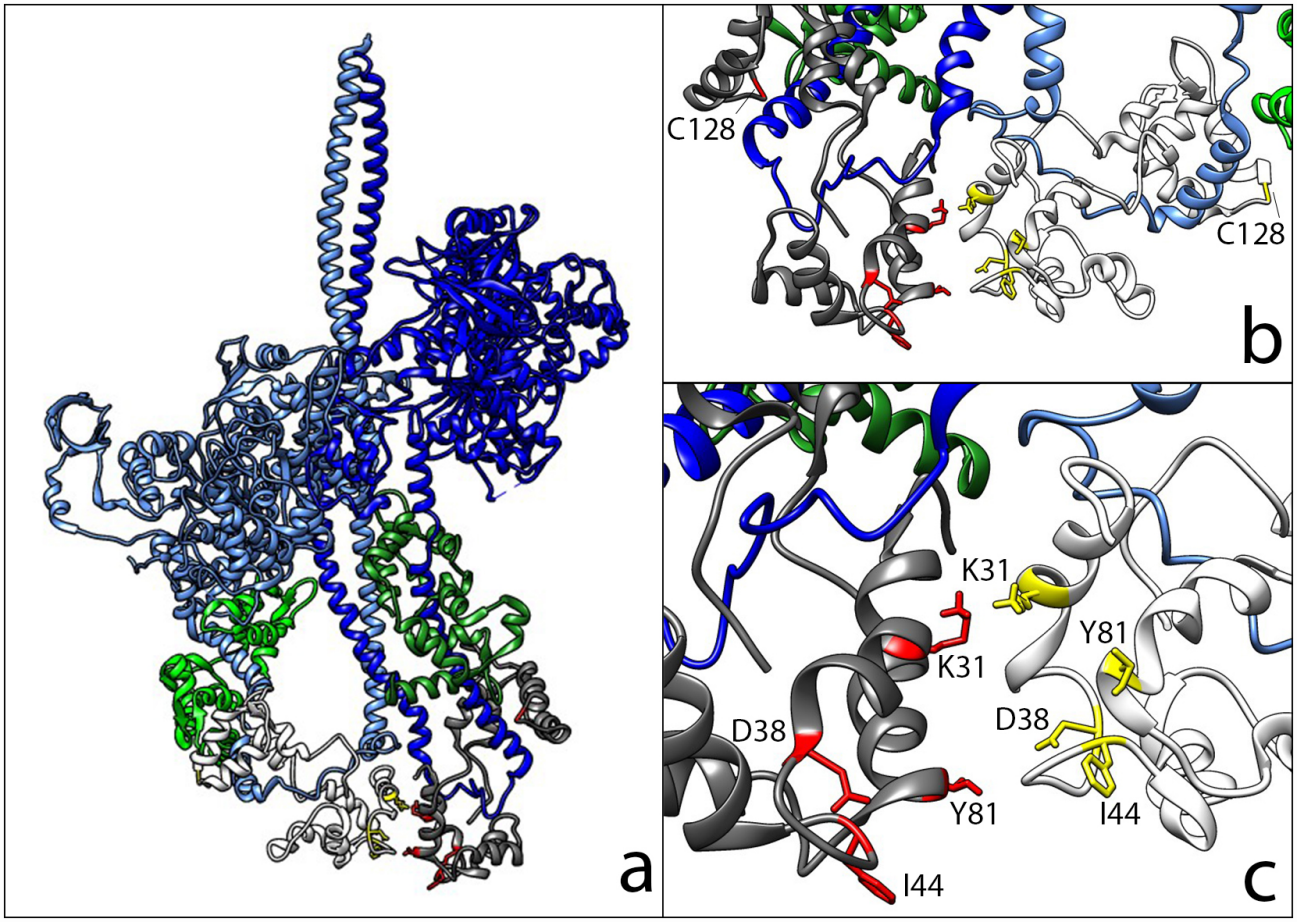
The structure of the tarantula IHM (3DTP) [9]. (A) The myosin heavy chain of the blocked head is light blue, and that of the free head is dark blue. The essential light chains are in light green and green, and the RLCs are in white and grey in the lower part of the structure. (B) The structure of the RLC domain as seen from the back of panel a, enlarged. Mutants are in yellow and red, C128 residues are highlighted. The first 50 residues in the N-terminus of the RLCs are not shown since they are very mobile and not present in the mouse RLC. (C) A closer view of the two RLC structures, as seen in panel b, with the mutants highlighted.

The first evidence for a structured state of the myosin heads in resting muscle came from the small angle x-ray diffraction pattern, where the strong myosin layer lines showed that a large fraction of the myosin heads were bound to the core of the thick filament in a helical array [4, 5]. The best model of the structure of the ordered array of myosin heads bound to the core of the thick filament is based on cryo-electron micrographs of myosin thick filaments from tarantulas and other invertebrates [9–11]. The structure, called the Interacting-Heads Motif (IHM) shows the two myosin heads of one molecule bound closely together and bent back on the rod portion of the coiled-coil tail and the core of the thick filament (Fig 1). The myosin head is composed of a large catalytic domain that contains the sites for binding actin and ATP, and a light chain domain formed by the binding of two small subunits, the essential light chain and the regulatory light chain (RLC). In the IHM there appears to be an interface between the two catalytic domains, an additional interface between the two RLCs, and an interface between the catalytic domain of the blocked head and the coiled-coil tail region. In addition, there are interfaces between adjacent myosin molecules both above and below in the helical array. The IHM was initially observed in two-dimensional crystals of a fragment of myosin from chicken gizzard [12]. It was later found in tarantula exoskeletal striated filaments, and has now been observed in a variety of invertebrate filaments, in vertebrate cardiac filaments and in isolated myosin molecules from vertebrate skeletal muscles [9–11, 13–17]. This leads to the hypothesis that the IHM is widely distributed in various muscles, and evolved early in the animal kingdom [11, 14, 18]. The IHM has also been observed by the polarization of bi-functional fluorescent probes on the RLC in skinned fibers from skeletal and cardiac muscles [19–21]. In skeletal fibers at physiological temperatures and lattice spacing, these probes show that virtually all of the heads are in the IHM [19].

In some of these myosins, scallop, smooth, tarantula, the IHM was shown to be associated with a highly inhibited ATPase activity, (turnover time of 30–60 minutes) [22–24]. These myosins all come from muscles controlled, completely or in part, through the thick filament. It is our proposal that this association also extends to vertebrate skeletal muscle, although with a faster ATP turnover time in the SRX of approximately 4 minutes.

The large amount of myosin in animals and humans leads to the conclusion that the equilibrium between the SRX and the DRX will play a role in whole body metabolism [25]. In the initial observation of the inhibited state in frog muscles it was noted that if all the myosins in the muscle had the ATPase activity observed in the test tube that the metabolism of the myosins alone would be 5 times greater than the metabolism of resting living muscle [1]. This shows that the vast majority of the myosins in the living muscle must be in the inhibited state as is also shown by fluorescent probes in skinned skeletal fibers in physiological conditions [19]. A high population of the SRX leads to energy economy, which is of obvious utility when energy/food is limited. From the point of view of a modern society, however, where energy can be harder to waste than to gain, a high population of the SRX can be counter-productive [25]. This leads to the concept that destabilizing the SRX would be a good target for increasing basal metabolism to treat weight-related diseases and Type 2 diabetes.

Several factors complicate the search for an assay that can measure the population of the SRX in a high-throughput screen, to find small molecules to disrupt the SRX and increase resting muscle thermogenesis. The most prominent of these is that in vertebrate striated muscles, the SRX has only been visualized easily in skinned muscle fibers. Our attempts to observe a SRX in purified proteins using fluorescent probes proved unsuccessful. With a lifetime of only 4 min. the kinetics of the chase experiment in fibers, are difficult to carry out in a plate reader. In the skinned fiber preparation there are other enzymes that use ATP, ion transporters, etc. There are also populations of myosin in the DRX and in damaged, unregulated fiber regions that are active with a very high ATPase activity. These factors complicate the direct steady-state measurement of ATPase activity as a method of monitoring the population of a state that has a very low ATPase activity. The signal that would be most useful in a screen is a fluorescent probe that has a spectral shift that was sensitive to the SRX-DRX transition.

Our study focused on the RLC as a site for spectroscopic labeling and sensing of the state of the myosin. The RLC has several advantages: 1. it is easily replaceable in skinned muscle fibers, 2. it may form one of the protein-protein interfaces that is important for the stability of the IHM complex [8–10], 3. it has a physiological site of regulation of the population of the IHM and SRX through its N-terminus phosphorylation [3, 8, 9, 26, 27]. Our first aim was to characterize the effect of a set of single cysteine RLC mutants, labeled with paramagnetic and fluorescent probes, on the stability of the SRX state. A second aim was to search for changes in the spectra of these probes that occur on the transition into the SRX. In particular, we were searching for a fluorescence signal that monitors the population of the SRX that would be a tool to scale the experiment up to the high-throughput level. The observations made here have provided information on the role of the proposed interface between the two RLCs in the stability of the SRX. We also describe one probe that provides a spectral signal that is amenable for measuring the population of the SRX in high throughput screening. Such screens can now find small molecules that disrupt the SRX, thus increasing metabolic rate to treat metabolic diseases in humans.

## Materials and Methods

### Animals

White New Zealand Rabbits, obtained from Charles River Laboratories Boston MA, were anesthetized with ketamine and xylazine followed by exsanguination and bilateralthoractotomy, protocols approved by the UCSF Institutional Animal Care and Use Committee, approval number AN108976-02. Psoas muscle skeletal fibers were harvested as described in [28]. The fibers were chemically skinned by immersion in buffers containing 50% glycerol, which renders them permeable to molecules such as the RLC.

### Chemicals and solutions

The Rigor buffer consists of 50 mM 3-(N-morpholino)propanesulfonic acid (MOPS), 120 mM potassium acetate, 5 mM MgCl_2_, 5 mM EGTA, 5 mM potassium phosphate, pH = 6.8, adjusted at RT. Relaxing buffers were obtained by addition of 4 mM ATP or 250 μM mantATP to the Rigor buffer. For The EPR experiments the relaxing buffer was obtained by adding a solution of 4 mM ATP and 40 μM blebbistatin to the rigor buffer. The RLCs were exchanged into fibers using exchange buffer containing 50 mM MOPS, 20 mM EDTA, 50 mM KAC, 10 mM potassium phosphate, pH7.0. Spectra were obtained at 24±2°C.

Blebbistatin ((±)-1-Phenyl-1,2,3,4-tetrahydro-4-hydroxypyrrolo[2.3-b]-7-methylquinolin-4-one), was obtained from Toronto Research Chemicals, Inc., 2 Brisbane Road, North York, ON M3J 2J8 Canada. The spin labels: 4-maleimido-2,2,6,6,-tetramethyl-l-piperidinyloxy (MSL), 4-iodoacetamido-2,2,6,6,-tetramethyl-l-piperidinyloxy (IASL) (1-oxyl-2,2,5,5-tetramethylpyrroline-3-methyl)methanethiosulfonate (MMTS) were purchased from Sigma Aldrich (USA) Chemical Co. All fluorescent probes: 3-(bromomethyl)-2,5,6-trimethyl-1*H*,7*H*-pyrazolo[1,2-*a*] pyrazole-1,7-dione (BIMANE); 7-Diethylamino-3-[*N*-(2-maleimidoethyl)carbamoyl]coumarin (MDCC); 2-(4'-maleimidylanilino)naphthalene-6-sulfonic acid (MIANS) were obtained from Sigma Aldrich (USA) Chemical Co. A stock of 20 mM of each was prepared in dimethylformamide and stored in the freezer at -20°C. P-6 spin columns were purchased from Bio-Rad. Thrombin was purchased from Sigma Aldrich (USA) Chemical Co. The proteins were visualized on 18% SDS gels obtained from NOVAX using either Instant blue stain (Expedeon) or silver stain that was prepared in the lab. Troponin C was kindly provided by Wen-Ji Dong (WSU). Other chemicals were purchased from Sigma (USA) Chemical Co.

### Molecular biology and purification

The mouse RLC DNA comes from a cDNA kindly provided by the Di Lisa’s Lab (University of Padova). cDNA was obtained from the tibialis anterior muscle of a 6 months old C57bl/6j mouse. The cysteines present in the sequence, positions C128 and C157, were mutated to alanines and the other cysteines were added using Qiagen Quickchange II mutagenesis kit (K5C, A6C, K31C, D38C, I44C and T81C). The RLC mutants were expressed overnight at 18°C in BL21 Rosetta cells as a N-terminal fusion protein with a histidine tag and thrombin protease site from a Novagen pET28 vector, kindly provided by Geeta Narlikar (UCSF). The supernatant was loaded on a Qiagen Ni-NTA agarose resin column in wash buffer (200mM NaCl, 50mM TES buffer, 20mM imidazole, 2mM MgCl_2_, 1mM dithiothreitol (DTT), 10% sucrose, pH 8.0) and washed extensively with the same buffer. Protein binding the resin was then washed with 10ml of wash buffer without DTT and eluted with elution buffer (wash buffer with 300mM imidazole, 100μM DTT and pH 7.6). The protein from the column was diluted to a concentration of approximately 100μM with dilution buffer (200mM NaCl, 50mM TES buffer, 2mM MgCl_2_, pH 7.6), labeled overnight at 4°C in the presence of 300μM of paramagnetic or fluorescent probes. The unreacted probe was quenched with 1mM DTT and, followed by digestion with thrombin if desired to remove the HIS-tag, see below. No DTT was added when spin probes were used for labeling as it reduces the label. Finally, the RLC was dialyzed in exchange buffer and used or stored at -20°C.

RLC-C5 and C6 were cleaved with thrombin immediately after labeling with the appropriate probe. The protein was left at 23°C for 20 minutes. One unit of thrombin and 200μM CaCl2 were added to the elution/dilution buffer, and the solution was left at 30°C for 3 hrs. The reaction was stopped by adding 300μM EDTA solution to remove the calcium. Depending on the amount of protein that was cut, the buffer was then exchanged into the fiber exchange buffer either by dialysis of by using P-6 spin columns.

### Exchanging RLC on muscle fibers

**Fig 2:**
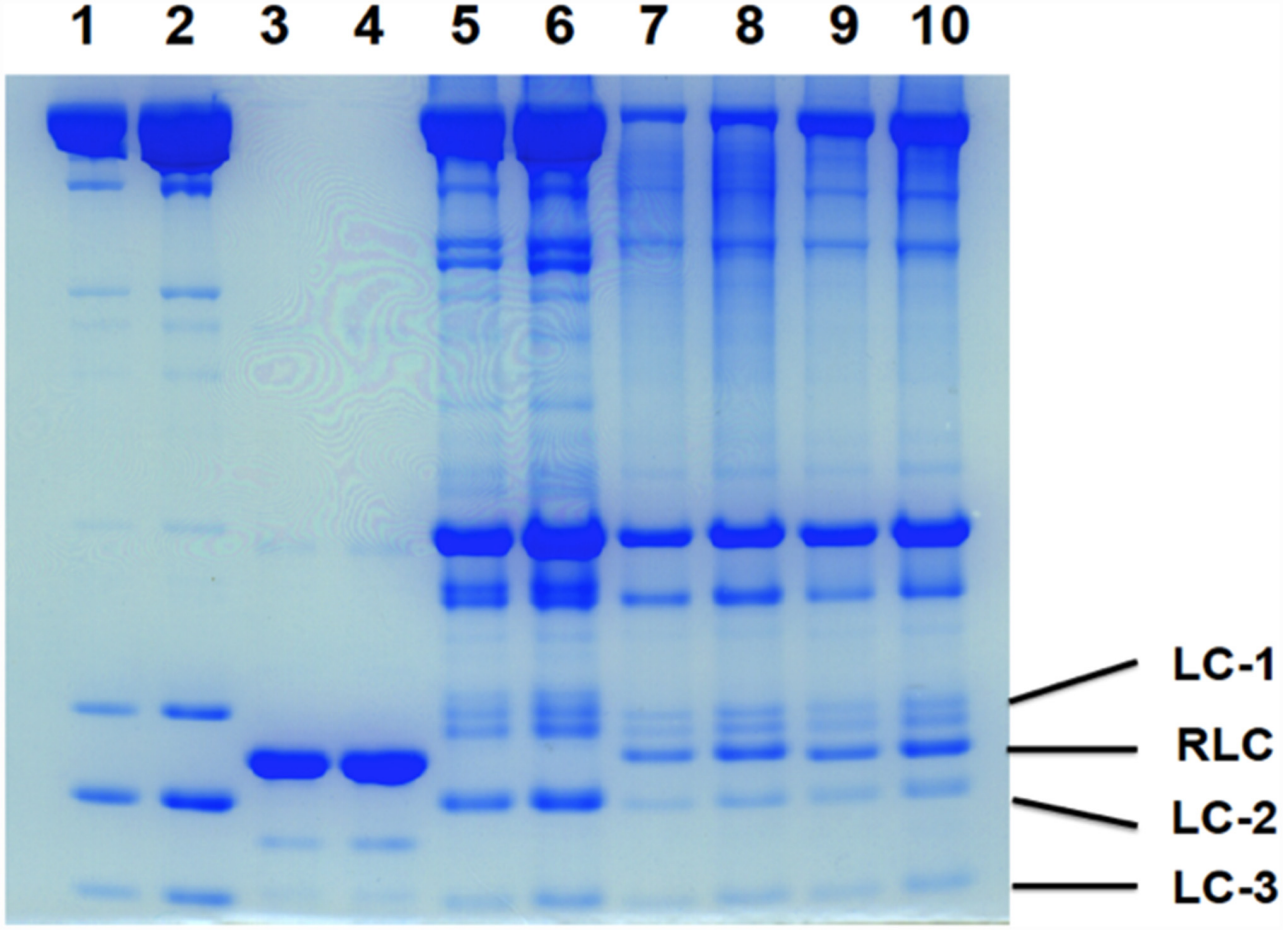
Gel electrophoresis of fibers and proteins used to quantitate exchange of mutant proteins into the fibers. Lane 1, 2 myosin; Lane 3,4 RLC-C5 uncut; Lanes 5,6, control unexchanged fibers; lanes 7,8 fibers exchanged with RLC-C44-MSL; lanes 9, 10, fibers exchanged with RLC-C44-MMTS. As can be seen in lanes 7–10 the intensity of the endogenous RLC (LC-2) is diminished with a new band showing the presence of the uncut mutant. Quantitation of the bands showed that the intensity of LC-2 is diminished by 68%, 71%, 73% and 82% in lanes 7–10 respectively. The intensity of the RLC bound to the fibers relative to LC-2 in the controls is 106%, 103%, 96% and 90% in lanes 7–10 respectively. Thus about 70% of the LC-2 in the preparations has been replaced by mutant RLC, with an additional 30% mutant bound non-specifically.

Small bundles of 8–10 rabbit psoas fibers, were washed with exchange buffer, and incubated in 100μM of labeled RLC for 1 hour at 30°C followed by 30 minutes at 30°C. The bundles were then incubated in a rigor solution containing 0.4 mg/ml Troponin C at 30°C for 1 hr. The bundles were then placed into a rigor solution with 50% glycerol, and stored at -20°C The extent of RLC exchange was determined by SDS gel electrophoresis and quantitation of the decrease in the endogenous RLC and the increase in the mutant RLC (Fig 2).

### Observations in plates

For observations in 384 well plates, fibers were minced with a scalpel, providing a preparation that consisted predominately of single fibers 50–200μm in length. The suspension of fibers was pipetted into wells in 384 well plates. The plates were imaged using a Nikon 6D epi-fluorescence microscope using excitation filters centered at 387nm and emission filters centered at either 440nm or 525nm. The images were analyzed using a macro that determined the intensities of fiber fluorescence in the two images allowing a calculation of their ratio.

### Spectroscopy

The major spectroscopic tool used here was a fluorescence microscope where fiber fluorescence could be measured quantitatively and artifacts due to movement of the fibers could be avoided, as described previously [3]. Single fibers were mounted in a simple flow cell and observed using quantitative epi-fluorescence with a Nikon 6D TE2000 microscope. For one sample that showed an important spectral shift, excitation and emission spectra were obtained using a Fluoromax-3 fluorimeter (Horiba Corp). Fibers were hung between two struts mounted in a 1cm cuvette. Comparison of intensities of 2 spectra obtained by this method was complicated by artifacts due to slight movements of the fiber that occurred during solution changes and due to photo decay. For the excitation and emission spectra shown, the difference in the absolute intensities of the spectra in different condition were determined by observing fibers in the fluorescent microscope using an emission filter that measured the peak intensity 660 to 670nm. This allowed us to measure the change in the peak intensity during the transition from rigor to relaxation. At the wavelengths used in the microscope, fiber autofluorescence was almost undetectable, less than 10 units, compared to a typical labeled fiber that had 2000 units. The autofluorescence was unchanged upon addition of ATP.

EPR measurements were performed with a Bruker EMX EPR spectrometer (Billerica, MA) as described previously [29]. Fibers with the exchanged RLC were aligned on an EPR flat cell and spectra obtained with fibers aligned either parallel or perpendicular to the magnetic field.

## Results

### IHM structure and how mutants were chosen

**Fig 3:**
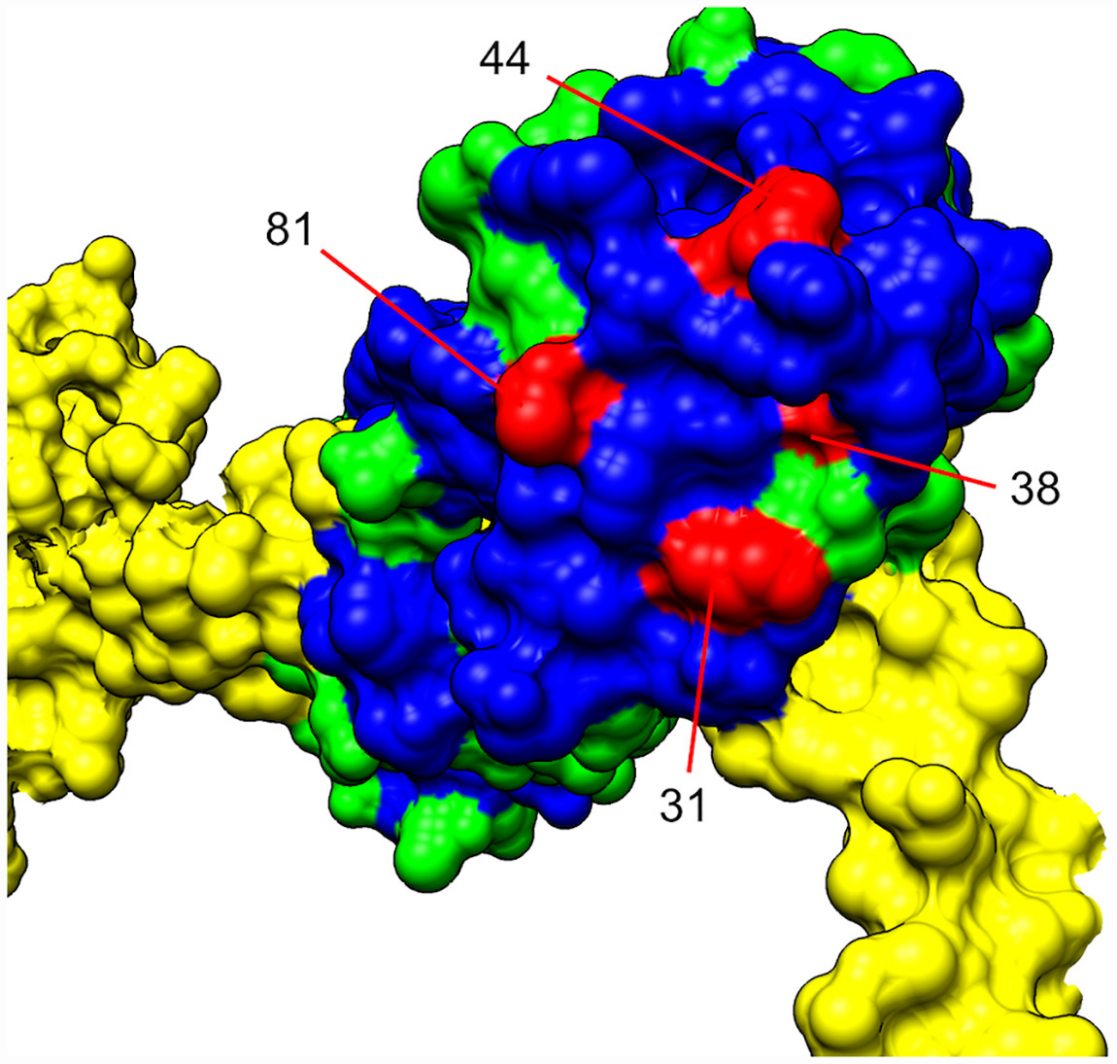
The structures of the N-terminal lobe of the RLC along with a portion of the heavy chain (in yellow) model are shown (picture adjusted from the 3J04 model of chicken smooth muscle myosin and RLC [35]). The non-conserved residues of the RLC are shown in green, the conserved residues are in blue, and 4 of the mutated residues are in cyan. The mutants, K31C, D38C, I44C and T81C are identified by their positions in the mouse construct used in our experiments. Conserved residues were identified by the chicken smooth muscle RLC and mouse skeletal muscle RLC alignment (S6 Fig).

The positions in which to insert the cysteines were selected based on sequence conservation and on the model of the IHM from the Protein Data Bank (3DTP) [9]. After completion of these studies, a model at higher resolution was published (3JBH), however the new model did not change any of the conclusions on the proposed interface that we had made [30]. A comparison between the sequences of tarantula exoskeletal muscle and vertebrate skeletal muscle (S1 Fig) shows that many of the conserved amino acids are clustered on the surface on the N-terminal lobe, close to the location of the RLC-RLC interface in the model of the IHM (Fig 3). The presence of a cluster of conserved residues on the surface of a protein suggests that they may have a role in protein-protein interactions. This cluster provided another method for choosing residues for probe sites. The model structure of the IHM suggests that the interface between the two RLCS is formed around the A helix, aa 30 to 40 (mouse sequence numbering), and the N-terminus of the D helix 76 to 88 [9, 10]. The ideal location to place probes to monitor the SRX will be at the border of the interface, will not destabilize the IHM and will sense changes in the SRX versus the DRX. Three sets of mutants were chosen, the first is in the very N-terminus, the second is in the surface region of the proposed interface. The third is in the C-lobe serving as a control. The first set has been designed with the idea that the N-terminus plays an important role in the stability of the IHM structure. A recent paper suggests that the N-terminus undergoes a structural change when phosphorylated [31–34]. The fact that phosphorylation affects the stability of both the SRX and the IHM, suggests a role of the N-terminus in the stability of the complex [3, 8, 9, 24, 27]. The extra N-terminus found on the invertebrate RLCs, compared to the mammalian RLCs, may explain the higher stability of the IHM in tarantula muscles, compared to rabbit muscles [8, 27]. The N-terminus is characterized by several positively charged residues. We explored the substitution of one of them with a cysteine in the K5C mutant and of a non-charged residues with the A6C mutant. The second set (K31C, T81C, D38C, I44C) is located in the proposed interface region according to the IHM structure (3DTP), and all were within or close to the cluster of conserved residues (Fig 3). The positions of mutants K5C AND A6C are not present in any crystal structure due to disorder. A mutant containing an endogenous cysteine, C128, acts as a control since it is far from the putative RLC-RLC interface.

### Measuring the stability of the SRX in skinned muscle fibers

**Fig 4:**
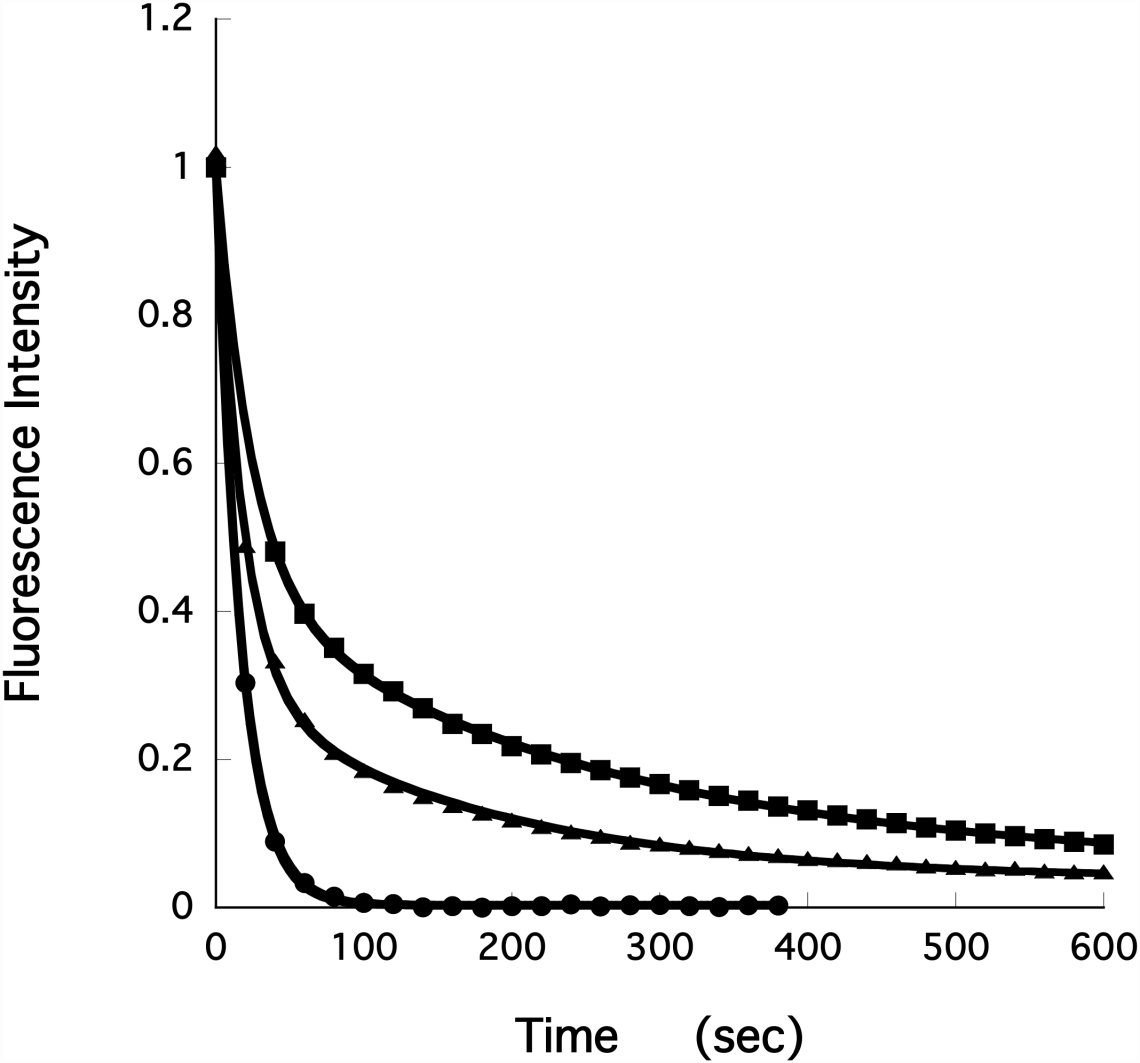
The intensity of the fiber fluorescence, relative to the pre-chase value, is plotted as a function of time during the chase phase of the single nucleotide turnover measurements. The fibers were previously relaxed in 250μM mantATP, and then chased with 4mM ATP. Fiber fluorescence decreases in two phases, a fast phase that is largely over in about 20–30 seconds, followed by a slow phase with a lifetime of minutes. The slow phase arises from the slow release of nucleotides by myosin in the SRX. The top trace (solid squares) shows a control fiber. The middle trace (solid triangles) shows a fiber exchanged with RLC-C6 labeled with MDCC. As shown the slower phase has a decreased population and a faster lifetime, indicating that the SRX has been partially destabilized. The lowest trace (solid circles) shows a fiber exchanged with RLC-C44 labeled with BIMANE. Here the slow phase is completely absent, indicating complete destabilization of the SRX.

Measuring the properties of the SRX in skinned fibers is complicated by the presence of other ATPases, ion channels, disordered myosin heads, etc. In steady-state assays, these faster ATPases dominate the slower SRX. To overcome this problem, we measured single nucleotide turnovers by first incubating the fiber in a relaxing solution that contained 250 μM mantATP. MantATP has been shown to bind to myosin with high affinity, and to be hydrolyzed with similar kinetics as ATP [36, 37]. The mantATP binds to the myosin, relaxing the fiber. In addition, it also binds non-specifically to other structures in the fiber. After incubation of the fiber in mantATP for several minutes, the fiber was chased with a relaxing solution containing ATP. The decay in fiber fluorescence occurs in several phases as mant-nucleotides are released from their sites and diffuse out of the fiber (Fig 4). There is a rapid decay that occurs in the first 20–30 seconds, followed by a slower decay that occurs with a lifetime that is approximately 4 minutes. The first phase consists of the diffusion of free nucleotides out of the fiber, which occurs in 10 seconds, the release of mantATP from most non-specific sites, which occurs quickly, and the turnover and release of mant-nucleotides from disordered myosin and from other enzymes, which occurs in 20 seconds or less. The slower phase, which has a lifetime of about 240 seconds, has been shown to be largely due to the slow turnover and release of mant-nucleotides from myosin that is in the SRX [3]. The data could be adequately fit by a function consisting of 2 exponential decays one with a short lifetime and one with a longer lifetime. The populations of the two phases were P1 and P2 and their lifetimes were T1 and T2.

### The effects of modified RLCs on the SRX

Fibers were exchanged with mutant RLCs and labeled mutant RLCs. Although the RLCs were cloned using DNA from mice, rat and rabbit RLCs have identical sequences. The fibers were incubated in mantATP and chased with ATP as described above. Fig 4 shows the fluorescence intensity during the chase phase. The fraction of the fluorescence intensity that decays with the longer lifetime, P2, provides a measure of the stability of the SRX. The lifetime of this phase, T2, is also an important property of the SRX.

The effect on the SRX depended on the location of the mutant and on the nature of the label. Fig 4 shows representative traces during the chase phase for three samples. For the control sample there is a fraction of the fluorescence that decays slowly during the chase, which represents 37% of the total fluorescence and has a lifetime of 248 seconds. This component arises from the slow release of nucleotides from myosin heads in the SRX. Although this component represents 37% of total fluorescence it represents a greater fraction of the myosin heads. There is a fraction of probes, ~40%, that are bound nonspecifically to the fiber, and are released quickly [3]. Thus only 60% of the total fluorescence arises from probes bound to myosin and the proportion of the myosin that is in the SRX is 62%. For fibers exchanged with RLC-C44-BIMANE, virtually all the fluorescence, 99%, is released in the fast phase. The population of the SRX in these fibers is close to zero. For fibers exchanged with RLC-C6-MDCC, there is a slow decay of fluorescence that represents 26% of the total with a lifetime of 172 seconds. Both the population and the lifetime show that the SRX in these fibers is destabilized relative to that observed in the control fibers. The fraction of fluorescence that decays slowly during the chase phase is proportional to the population of the SRX. As can be seen by the values of P2 summarized in Table 1 (bar graph S2 Fig) the mutations alone have a minimal effect on the stability of the SRX. For only one of the mutants, RLC-C38, did the mutation alone destabilize the SRX, decreasing the value of P2 by 35%. The D38C mutation is in an aspartic acid that coordinates to the divalent cation that is bound to an EF hand in the N-terminal lobe of all RLCs. In some cases, the attachment of probes to the single cysteines decreases the population of the SRX. Some of the probes are small, MMTS and BIMANE, some are intermediate, MSL and IASL, some are large but attached by a flexible leash, MDCC. One is large but not flexible, MIANS, and would be expected to provide the most interference. The structures of the probes are shown in S3 Fig. The pattern of interference produced by the attachment of probes could provide information on the role of the various sites in the proposed interface formed by the two RLCs in the SRX. Mutant C128, one of the native cysteines, is used as a control since it is in the C-terminus lobe, far from the putative interface. None of the probes attached to it affect the population of the SRX, as expected for a control. Two of the probe sites, D38C and I44C cannot tolerate even the smallest of the probes, MMTS or BIMANE. These sites are located in the region of a structural motif known as an EF hand, which binds divalent cations, either Mg^2+^ or Ca^2+^. In fact, the mutant D38C was the site that produced the greatest effect on the value of P2 even in the absence of an attached probe as discussed above. The second mutation in this region, I44C, is also in the EF hand structure. Although it does not coordinate the divalent cation and is not completely conserved, it forms part of the structural motif that binds the divalent cation, see S1 Fig. These observations attest to the importance of the presence of the divalent cation in the stability of the SRX. The sites K31C, and T81C are within both the cluster of conserved residues, and the proposed interface. Both of these sites tolerated the smallest probes or the flexible probes, while larger and stiff probes partially affected the stability of the complex (Table 1 and S2 Fig). The sites on the N-terminal region tolerated many but not all of the probes, with little correlation between probe properties and their effect. In general, the destabilization of the population of the SRX was accompanied by a corresponding decrease of T2. However, there was a lot of scatter in the data. Although the data provide additional support for the hypothesis that the inhibited heads of the SRX are in the IHM, they do not prove this possibility. Furthermore, the supposition that there are also myosin heads that have an inhibited ATPase activity but are not in the IHM cannot be ruled out by our data.

**Table 1:** Fraction of the Fluorescent Nucleotides Released Slowly during the Chase Phase.

LABEL	C5	C6	C31	C38	C44	C81	C128
NONE	0.26 ± 0.02	0.27 ± 0.04	0.28± 0.02	0.21 ± 0.02	0.31 ±0.02	0.31 ± 0.02	0.31 ± 0.02
MMTS	0.23 ± 0.03	0.25 ± 0.02	0.29 ± 0.02	0.10 ± 0.03	0.07 ±0.02	0.24 ± 0.02	0.28 ± 0.05
IASL	0.15 ± 0.04	0.10 ± 0.02	0.31 ± 0.02			0.19 ± 0.04	0.29 ± 0.01
MSL	0.19 ± 0.04	0.25 ± 0.02	0.20 ± 0.01			0.27 ± 0.02	0.31 ± 0.01
BIMANE	0.22 ± 0.03	0.16 ± 0.03	0.36 ± 0.04	0.08 ± 0.02	0.03 ± 0.03	0.32 ± 0.02	0.29 ± 0.03
MDCC	0.25 ± 0.02	0.26 ± 0.01	0.32 ± 0.01			0.36 ± 0.02	0.28 ± 0.03
MIANS	0.13 ± 0.03	0.20 ± 0.02	0.10 ± 0.04			0.17 ± 0.04	0.34 ± 0.02

The fraction of the nucleotides that are released in the slow phase, measured by the mantATP chase experiments as shown in Fig 4, is shown for various preparations of exchanged fibers. The unexchanged control fibers had a fraction P2 = 0.32. Fibers that went through the exchange protocol but without exchange of the RLC had similar values of P2. The fraction of myosin heads in the SRX is equal to approximately 1.7 x P2. Data are mean +/- SEM as determined from 5–10 individual measurements.

### Spectra of fluorescent probes on the RLC

Fluorescent probes and paramagnetic probes are able to sense changes in the surrounding environment but do so in different ways. Paramagnetic probes can sense differences in terms of the mobility of the probe and orientation of the sample. Fluorescent probes sense the hydrophobicity of the environment, characterized by the side chains of the residues that are close to the probe in the protein structure. We were able to detect spectroscopic changes using both fluorescent and paramagnetic probes.

**Fig 5:**
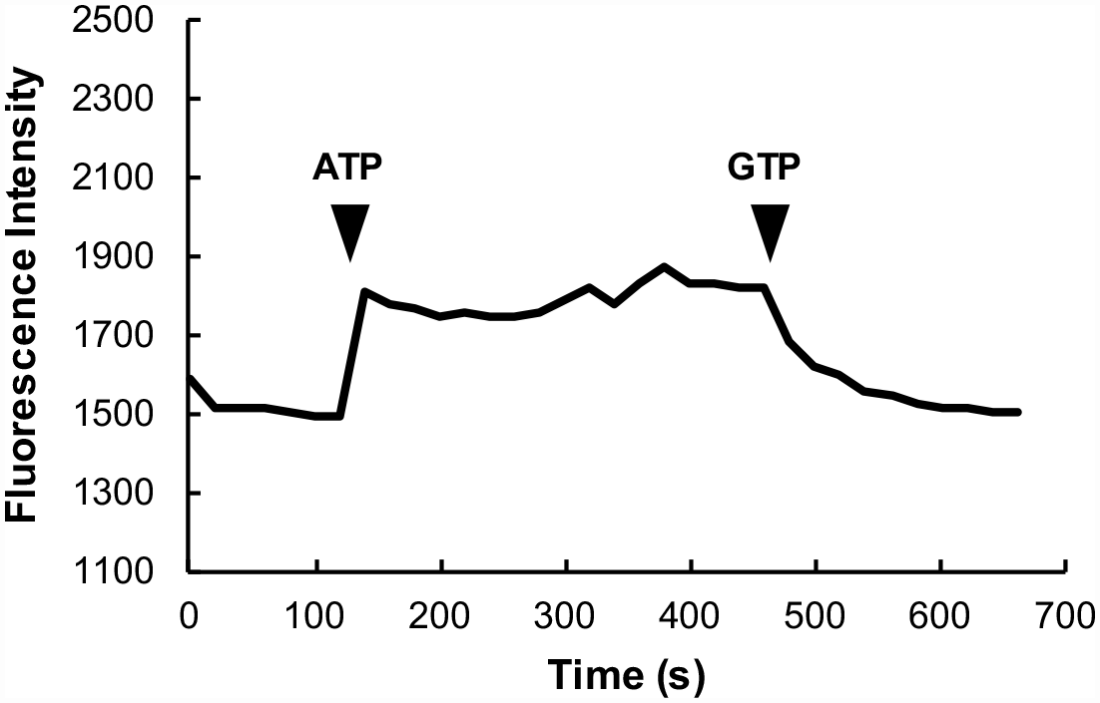
The intensity of the fluorescence of fibers exchanged with RLC-C31-MDCC is shown as a function of time. The fiber starts in rigor. At 120 seconds ATP is added to relax the fiber and populate the SRX. At 450 seconds the ATP is replaced by GTP, which keeps the fiber mechanically relaxed but de-populates the SRX.

The probes described here are all environmentally sensitive, e.g., their quantum yields are altered by changes in the polarity of the surrounding medium. All of the probes were examined for spectral changes in a protocol that typically started in rigor, ATP was then added to populate the SRX followed by GTP to populate the DRX (Fig 5). It is known that in rigor fibers all myosin heads are attached strongly to actin and thus the population of the SRX is zero [3]. The chase experiments have shown that in the ATP relaxed state approximately 60% of the myosin heads are in the SRX, leaving the remaining 40% in the DRX [3]. Studies using x-ray diffraction found that myosin heads were ordered by binding to the core of the filament in ATP, but that they were more disordered in fibers relaxed by GTP [7]. We showed earlier that in fibers relaxed in GTP that the population of the SRX and T2 were decreased by a factor of 3, relative to that observed for fibers relaxed in ATP [3]. Thus in the presence of GTP, fibers are mechanically relaxed with 90% of the myosin heads in the DRX and 10% in the SRX. Thus on the addition of ATP to rigor fibers 50–60% of the myosin heads make the transition from a rigor state to a more ordered SRX, and upon addition of GTP, two thirds of these return to a disordered, but still relaxed state.

The changes in fluorescence are shown for MDCC attached to RLC-C31 in Fig 5 and for selected mutants in Table 2. As shown in Fig 5 fluorescence intensity increases by about 15% in the transition from the rigor state to the ATP relaxed state and then decreases by a similar amount in the transition to the GTP relaxed state. The first conclusion is that the spectral intensity is similar in rigor and in fibers relaxed in GTP. This was generally observed for most of the probes that gave spectral changes (see Table 2). This suggests that the increased fluorescence intensity comes from the transition of myosin heads from a disordered state, either rigor or the DRX, into the SRX, where the RLC-RLC interface is formed in the IHM. Although the magnitude of the change, 15%, is small the actual change for each head making the transition into the SRX is larger when corrected for non-specific binding. Thus the actual change in the intensity of a labeled head is 25%.

**Table 2:** Changes in the Intensity of the Fluorescence.

LABEL	TRANSITION	C5	C6	C31	C81
BIMANE					
	RIGOR>ATP	-8% ± 2%	-5%	+15 ± 3%	+14 ± 3%
	ATP>GTP	<2%	+2%	-25 ± 3%	-17 ± 5%
MDCC					
	RIGOR>ATP	+18% ± 4%	+21%	+15 ± 4%	<2%
	ATP>GTP	-19% ± 4%	-11%	-14 ± 4%	<2%
MIANS					
	RIGOR>ATP	-8% ± 2%	-6 ± 2%	<3%	<3%
MIANS	ATP>GTP	+3%± 2%	+5 ± 1%	<3%	<3%

Changes in the intensity of the fluorescence on the transition from the rigor solution to the ATP relaxing solution and for the further transition from ATP relaxing solution to a GTP relaxing solution. There were no fluorescence changes for RLC-C38-BIMANE AND RLC-C44-BIMANE, and these constructs were not pursued further. Data are mean +/- SEM as determined from 4–6 individual measurements.

Changes in fluorescence intensity similar to those shown in Fig 5 were also observed in fibers exchanged with RLC-31-BIMANE, RLC-C81-BIMANE, RLC-C5-MDCC and RLC-C6-MDCC (Table 2). In addition, several samples showed a decreased intensity, on going from rigor to ATP-relaxed, although the changes were smaller, and not always reversed by GTP. The observation of a number of spectral changes, together with the inhibition of the SRX by some probes, suggests that the region targeted here, the putative RLC-RLC interface and the N-terminal end are both involved in the formation of the SRX.

A number of labeled mutants did not show spectral changes. In some cases, this is not unexpected. Probes attached to RLC-C128, far from the interface showed no changes, with the exception of the sample with BIMANE, which showed a small change, on rigor to ATP only. Probes attached to RLC-C38 or RLC-C44 showed no changes (Table 2), as expected because exchange of these labeled mutants into fibers completely destabilized the SRX. Mutants labeled with MIANS showed few spectral changes, but MIANS also inhibited the formation of the SRX, due to its size and rigid attachment to the protein. In interpreting these results it is important to consider that the structural model used to pick probe sites was not from a mouse RLC, and the exact environment of a given probe in a mouse protein is difficult to determine.

### EPR spectra

Electron Paramagnetic Resonance (EPR) spectroscopy can monitor the orientation and mobility of paramagnetic probes. Biological samples are usually not paramagnetic, thus, to do EPR measurements a paramagnetic probe has to be added to the sample. The unpaired electron has a magnetic moment, which gives rise to a spectrum. The mobility of the probe can be determined from the spectra and is related to the topology of the surrounding environment. In addition to mobility, an oriented sample such as muscle provides a good system to measure the orientation of the probes. The spectrum of a probe in an EPR experiment is sensitive to the angle between the magnetic field direction and the principal axis of the probe. A probe bound to the RLC will have a helical order if the RLC is bound to the core of the thick filament. A comparison between the spectra obtained with the orientation of the fibers, parallel and perpendicular to the magnetic field can give information on the orientation distribution of the probes.

Bundles of fibers were dissected and placed in a flat cell that was observed with the fiber axis either parallel or perpendicular to the magnetic field. Spectra were obtained in rigor or in ATP with the SRX stabilized by addition of blebbistatin. Blebbistatin was needed to maintain the SRX during the long incubations required for data collection, 10–20 minutes [29].

**Fig 6:**
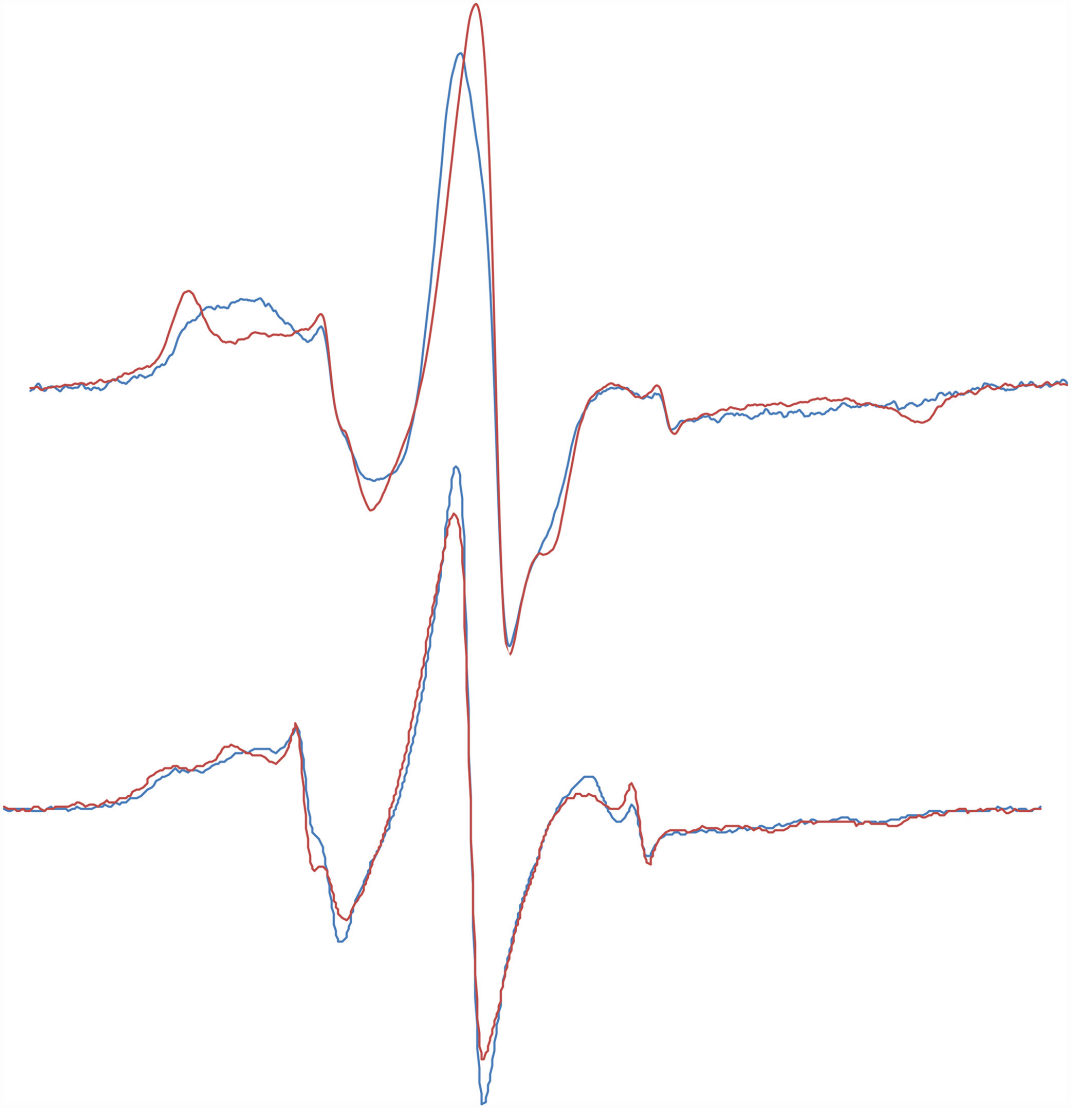
EPR spectra of fibers exchanged with RLC-C31-MMTS with the fiber axis either parallel, blue thin line or perpendicular, red thick line, to the magnetic field. The derivative of absorption is shown as a function of the magnetic field strength. The fibers are in a relaxing solution, ATP plus bebbistatin, above or in rigor, below. The difference between the parallel and perpendicular spectra in the top figure show that the paramagnetic probes are oriented in the SRX. In rigor there is little orientation. The center-fields of the spectra are 0.3490 T and the sweep width is 10 mT.

One mutant-probe pair gave appreciable spectral changes between rigor and the ATP relaxed state, RLC-C31-MMTS (Fig 6). This probe was oriented in the ATP-blebbistatin state, with more probe immobilization relative to rigor. The probe on RLC-81-MMTS was also more immobilized in the ATP-blebbistatin state than in rigor (S4 Fig). MSL was very immobilized on both RLC-31 and RLC-81, but showed no changes in orientation or mobility, see S4 Fig. IASL was more mobile on all sites, and did not show spectral changes between parallel and perpendicular or between rigor and ATP-blebbistatin. Probes on RLC-C5 and RLC-C6 showed two components one immobilized as expected for attachment to secondary structure and a second with more mobility. Fibers exchanged with RLC-C5-MMTS were more immobilized in ATP-blebbistatin than in rigor (S5 Fig). As expected probes on RLC-C128, RLC-C38 or RLC-C44 did not show spectral changes in different myosin states as also seen with fluorescent probes.

### Developing a signal for high throughput screens

**Fig 7:**
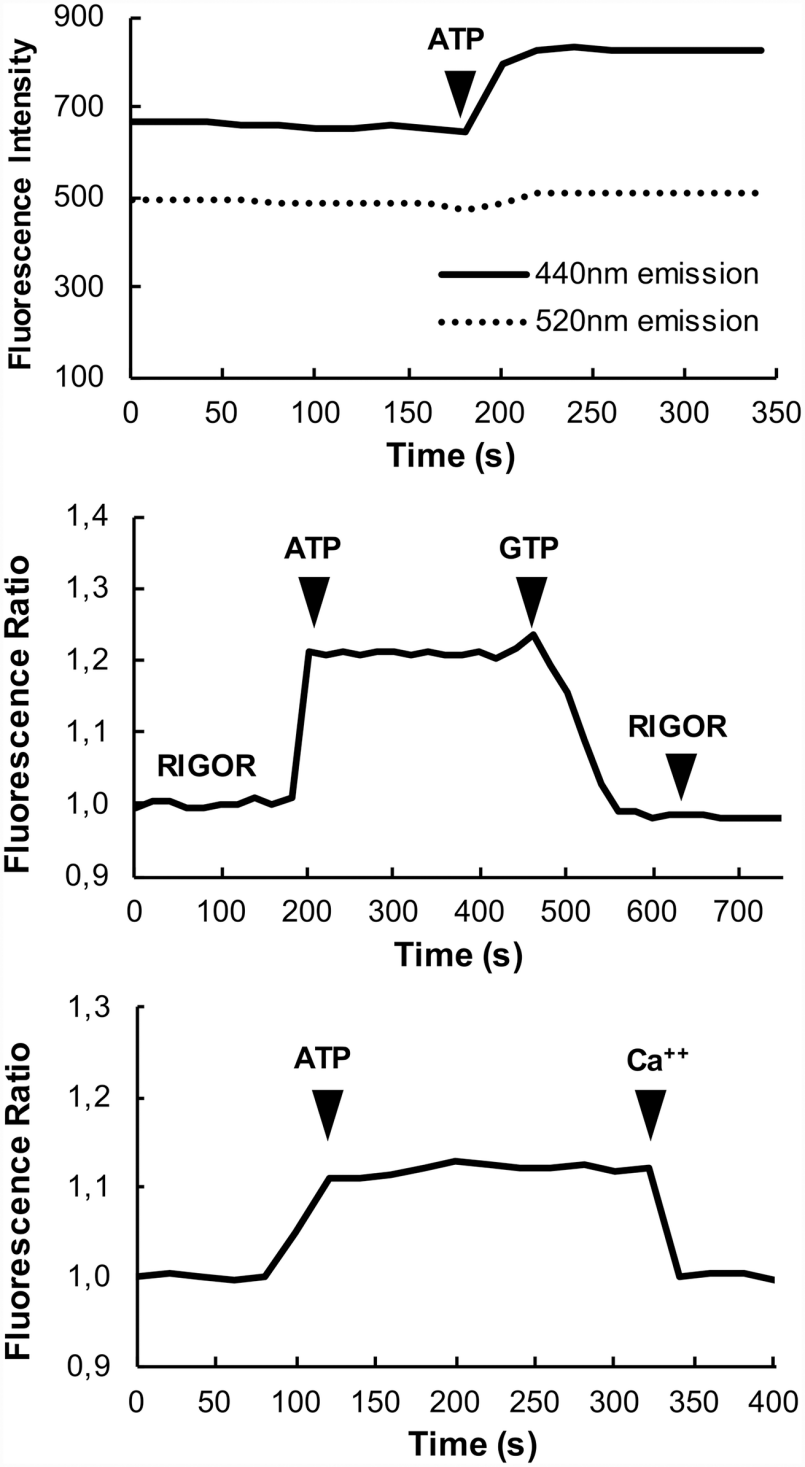
*Top panel*: Measuring two regions of the emission spectrum of RLC-C5-MDCC. Fibers were excited with a 387/11nm filter, and emission was observed on the blue side of the emission peak (440/40nm) and on the red side of the peak (525/50nm). The fiber starts in rigor. The figure shows that the response to the addition of ATP is much greater at the shorter wavelength. *Middle panel*: The ratio of the two signals over a wider time span, showing the response of the signal to the addition of first ATP, which is then exchanged for GTP and finally back to rigor. *Bottom panel*: showing that the ratio signal also responds to the full activation of the fiber, a condition known to destabilize the SRX [25]. In the middle and lower panels, the data have been normalized so that the rigor ratio is 1.

**Fig 8:**
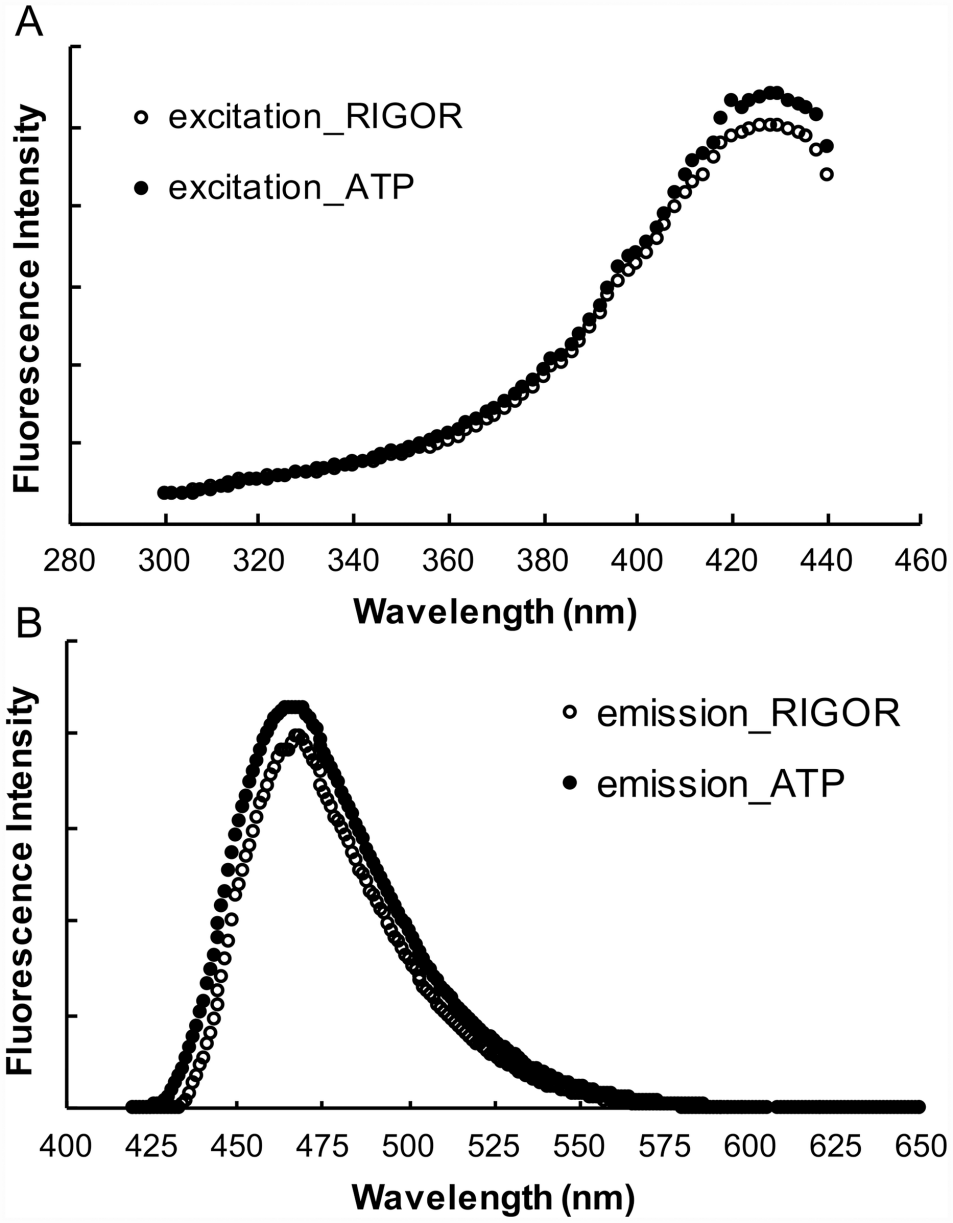
Excitation (panel *A*) and emission (panel *B*) spectra of RLC–C5–MDCC exchanged into fibers. Fibers were mounted in a fluorescence cuvette as described in Methods and observed first in a rigor solution followed by a relaxing solution. Intensities are shown in arbitrary units. For excitation spectra the emission was observed at 525nm, and for emission spectra the fibers were excited at 387nm. As can be seen there was a shift in the emission spectrum to shorter wavelengths in the relaxing solution. There was also an increase in absolute intensity, whose magnitude was determined by quantitative epi-fluorescence microscopy as described in Methods. There was no change in the shape of the excitation spectrum.

A major goal of this research was to find a change in the fluorescence spectra that would allow us to measure the stability of the SRX in a manner that is amenable to high throughput screens. As the only reliable measurements of the stability of the SRX require observations of muscle fibers these high throughput screens will be carried out by observing fibers minced with a scalpel, and pipetted into wells in 384 well plates. As each well will have different numbers of fibers, the excitation is not uniform and the positions of the fibers may change during the assay, reliable observations require a signal that does not depend on the amount of fibers in the field of view. A standard method for obtaining such a signal is to use the ratio of two different intensities that respond differently to changes in the population of the SRX. In some cases, probes show a blue shift e.g. a shift in the emission maximum to shorter wavelengths upon an increase in quantum yield; both due to a decrease in the polarity of the surrounding environment of the probe. The presence of a blue shift in our fibers was measured by using filter sets to observe intensities on the short and long sides of the emission maximum. In a survey of the probe spectra that showed changes in quantum yield we found only one signal, the fluorescence intensity of RLC–C5–MDCC exchanged into fibers that showed an appreciable blue shift, see Fig 7 for fluorescence intensities measured in the microscope. The emission spectra of the other probes changed in intensity but not in shape. The fibers were excited using an excitation filter centered at 387nm and observed using emission filters centered at 440nm and at 520nm. The blue shift caused the increase in intensity at the short wavelength side of the spectral peak to be larger than the change in the long wavelength side of the peak (Figs 7 and 8). By taking the ratio of the two spectral components we obtained a signal that was independent of the number of fibers in the field of view and was also not sensitive to photo bleaching of the probe. The ratio signal obtained in our standard experiment is shown in Fig 7. The ratio of the two intensities increases by 18% in the transition from rigor fibers to fibers relaxed in ATP and decreases by a similar amount upon subsequent relaxation of the fibers in GTP. The population of the SRX is known to be decreased significantly in the presence of GTP [3, 7]. The bottom panel shows that the ratio also responds to activation of the fibers, a condition in which the population of the SRX is also decreased [25, 38]. The average change in the ratio between rigor and relaxed fibers was 15 ± 3% (SEM n = 20). The ratio is independent of the amount of material being observed, and provides the long sought for signal that can be used to carry out high throughput screens in 384 well plates.

To further explore the spectral changes for this important probe we measured the excitation and emission spectra as a function of the wavelength, see Fig 8. There is both an increase in the magnitude of the intensity and a shift to shorter wavelengths upon the transition from rigor to relaxing solutions. The spectral changes shown in Fig 8 can be compared to those obtained by the microscope shown in Fig 7 by integrating over the regions selected in the microscope. Emission at the short wavelength 420–460nm increased by 38% while the longer wavelength 500–550nm increased by 24%. The ratio between short and long wavelengths increased by 11%, which is not far from the average seen in the microscope of 15%.

**Fig 9:**
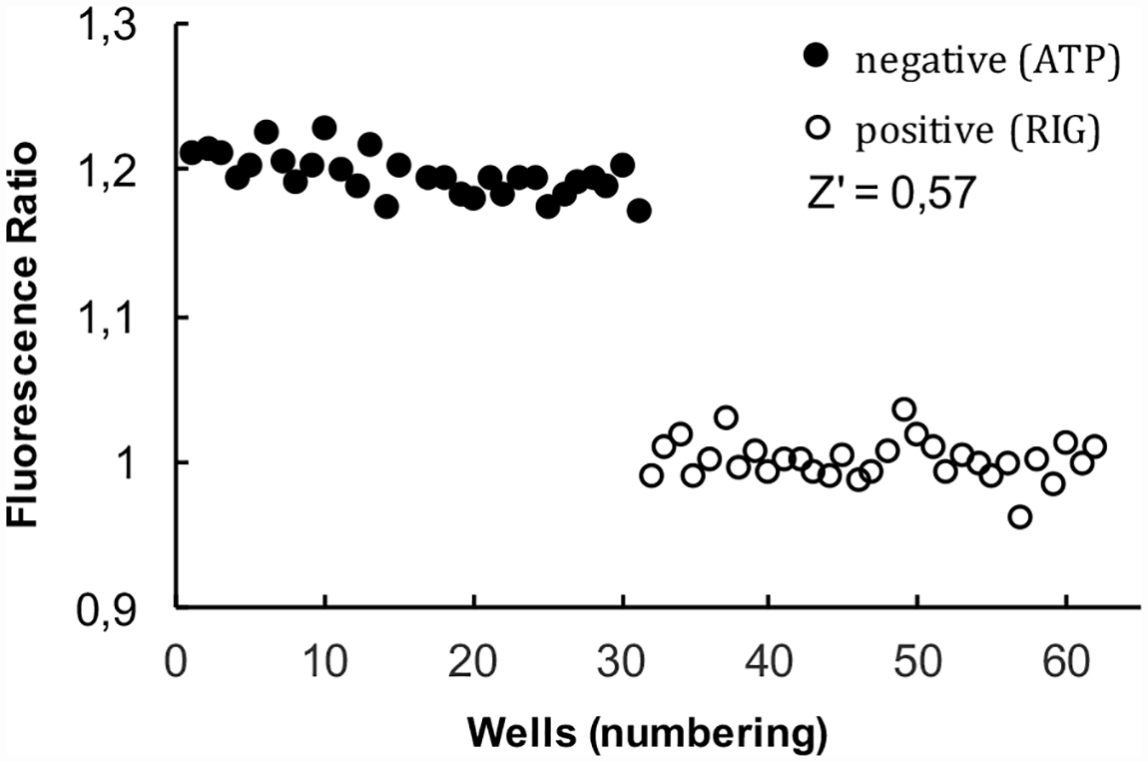
Measuring the SRX in a plate reader. Fibers labeled with C5-MDCC were minced and pipetted into wells of a 384 well plate. Images were obtained using quantitative epifluorescence at both short and long emission wavelengths. Images were analyzed to determine fiber fluorescence and the ratio of intensities is shown. The first 32 wells show data obtained in the presence of ATP. The average ratio is 1.195 ± 0.014 (st dev), characteristic of the SRX. The next 32 wells show data obtained in rigor, where the ratio is normalized to 1.0 ± 0.013 (st dev). The Z’ factor calculated from the data was 0.57 indicating that a more extensive screen using these methods is realistic.

Fig 9 shows the results obtained for minced fibers in a 384 well plate reader. Fibers were pipetted into the wells and incubated in either relaxing solutions or rigor. The number of fibers per well varied from approximately 20–50. As expected the ratio is higher in the relaxing solution. The difference in the ratio is greater than the standard deviations. The ability for the data to function in an extensive screen can be quantitated by a factor known as Z’:

$$Z^{\prime }=1-\frac{3\left(\sigma^{R}+\sigma^{T}\right)}{\left|M^{T}-M^{R}\right|}$$

Where σ^T^ and σ^R^ are the standard deviations and M^T^—M^R^ are the means of the data in relaxing and rigor solutions respectively. A value of Z’ between 1 and 0.5 indicates that screens would be good, values below 0.5 show that screens would be difficult and values below 0 show screens would be impossible. The value for the data of Fig 9, Z’ = 0.57, demonstrates that a screen using the C5-MDCC signal is possible.

## Discussion

Energy management and energy economy are important elements in evolution. To survive, animals, need to move, and to do so muscles have been developed. Muscles are powerful tools optimized for moving, mating, fighting, hunting and escaping. Because of the complex functions of muscle tissue, it is logical to think that it needs multiple modes of regulation. In particular, an important function is to manage energy expenditure. The SRX provides a mechanism whereby striated muscle can achieve higher energy economy. Our goal in this study was two-fold. First was to understand the role of the RLC in the structure of the IHM. Second was to find a signal that would allow the use of high throughput screens to search for small molecules that would change the stability of the IHM and SRX, and thereby alter the energy economy of muscle to influence whole body metabolism.

The mutations alone had a small effect on the stability of the SRX as measured by our chase experiments, see Table 1 and S2 Fig. A single mutation would not be expected to have a large effect on the stability of the SRX as the interfaces that form this complex involve extensive areas, and the perturbation in energy caused by changing one amino acid to cysteine will be small. Only one mutation had a sizable effect, and is discussed below. Addition of probes to the sites on the RLC exerted larger changes to the stability of the SRX. The pattern of inhibition was consistent with the hypothesis that the patch of conserved residues, which occurs in the region identified by cryo electron microscopy is in fact the interface. This is also supported by the pattern of probes showing changes in fluorescence spectra.

The best evidence for the RLC-RLC interface is the juxtaposition of these subunits in the 3D electron microscopy structures [9, 30]. Although the data described above support this hypothesis these data must be interpreted with caution. The inhibition of the SRX could also be caused by local unfolding of the protein. We tried to explore this by observing circular dichroism spectra of the labeled light chains, however in the absence of a heavy chain the RLC was not sufficiently well folded to obtain meaningful data. An RLC-RLC bond has never been observed in solution. In spite of extensive kinetic investigations a state with an inhibited ATP turnover has not been observed in skeletal myosin or heavy meromyosin [39]. We think that the RLC-RLC bond is weak, and only plays a major role in the context of the formation of the IHM array, where many myosins operate cooperatively to provide stability. Thus the most conservative interpretaton of the data provided here is that perturbations to this region of the N-terminal lobe of the RLC can alter the SRX.

The data also identify two regions of the RLC as playing important roles in the stability of the SRX, the binding site for divalent cations and the N-terminal end, see Fig 4, Table 1 and S2 Fig. The RLC has one site that binds both Mg^++^ and Ca^++^ ions. Although the site primarily binds Mg^++^, during periods of intensive activity Ca^++^ will replace a fraction of the Mg^++^ [40]. The role of this site remains undefined. Complete loss of the ability to bind cations in skeletal muscle decreases maximum tension by modulating the kinetics of cross bridge attachment and detachment [41, 42]. In cardiac muscle, loss of binding leads to cardiomyopathies [43]. Here we show that alterations of this site lead to a strong destabilization of the SRX. Although it is not clear from the structure whether it is involved directly in the proposed interface, it forms one side of the patch of conserved residues discussed previously. One of the amino acids changed, aspartate 38, would strongly affect binding of a cation as it is involved in the binding. The other amino acid, isoleucine 44, points away from the bound cation and into a position where it would communicate with the rest of the interface. Our results show that the divalent cation is involved in the stability of the SRX. This conclusion, along with the fact that Mg^++^ can be replaced by Ca^++^ during muscle activity [40], suggests that the divalent cation may regulate the SRX.

The N-terminus has been shown to be important in the formation of the IHM in smooth muscle [44], and we here extend this result to skeletal muscle. Probes placed on amino acid 23 in smooth muscle myosin fragments show changes in the conformation of this region upon phosphorylation only in the presence of nucleotide and only in heavy meromyosin and not in subfragment-1 [45]. These observations show that this region of the peptide, which lies between the N-terminus and the putative interface changes in response to the formation of the RLC-RLC interface. Providing further support for the conclusions drawn here. The role of the N-terminus in the formation of the SRX provides support for a structural model of a conformational change in the N-terminal region of the RLC upon the transition from the dephosphorylated form (presumably the SRX conformation) to the phosphorylated form (presumably in the DRX) [27, 31–34, 46]. This involves a transition from a compact region lacking secondary structure to an alpha-helical region that is more solvent exposed. Our data also show that there is a tendency for the probes in the N-terminal region to be less solvent exposed in the SRX, which provides further support for the model.

The observation that the addition of a probe to a site within the RLC-RLC interface destabilizes this interface provides a proof of concept that the binding of small molecules could decrease the stability of the SRX. Although the structure of the IHM involves multiple interfaces the steric disruption of one of these interfaces by an attached probe is sufficient to destabilize the entire complex. Because full inhibition was found for some samples, in which only 50% of the RLC's were exchanged, the data further show that the sub-stoichiometric binding of an inhibitor can destabilize the SRX completely. The SRX is completely destabilized in spite of the fact that 25% or more of the myosins in the array have not been modified. Thus destabilization of one molecule also destabilizes adjacent molecules, and the myosin heads in the array are interacting in a cooperative fashion. Destabilization of a fraction of them destabilizes the entire array, which then acts as a unit. A variety of data support the idea that the energetics of the formation of the IHM is cooperative. A cooperative inhibition of the SRX has previously been observed for RLC phosphorylation where the maximum inhibition is achieved when less than 50% of the myosins are phosphorylated [3]. Some of the most compelling evidence for cooperativity comes from small angle x-ray diffraction measurements in which the muscle fibers are activated at longer sarcomere lengths, where a large fraction of the myosin heads are not in the overlap zone [5, 6]. Even though half or more of the myosin heads have no thin filaments to interact with, upon activation all of the myosin heads become very rapidly disordered. This observation shows that there has been a signal passed from the myosin heads in the overlap zone to other distant myosin heads causing them to become disordered. A cooperative mechanism has also been proposed for the activation of tarantula thick filaments by phosphorylation [8, 47].

In the presence of a magnetic field an unpaired electron gives rise to a spectrum consisting of three lines. The splitting between the spectral peaks depends on the angle between the principal axis of the probe and the magnetic field: the wider the spectral splitting the smaller the angle. A comparison of the spectra taken with the long axis of the fiber parallel or perpendicular to the magnetic field provides information on the orientation distribution of the probes. Such a comparison is shown in Fig 6, for fibers exchanged with RLC-C31-MMTS, the only sample to show good orientation. The splitting between the high-field and low-field peaks is less in the parallel spectra showing that the long axis of the probes is oriented predominantly more perpendicular than parallel to the fiber axis. More quantitative interpretation of this orientation is difficult as the two RLC's of a given myosin molecule have two different orientations, and in addition we do not know how the probe is oriented relative to the protein. However, we note that oriented probes even in muscle fibers are rare. In contrast for these fibers in rigor there was no preferred orientation of the probes relative to the magnetic field, shown in Fig 6. In the modeled tarantula IHM, the residue number 31 seems to be in an ideal position to sense the changes in the conformation of the complex. The original lysines are right at the interface between the two RLCs, facing each other. The surface is not tight and a small probe may fit in the empty space, which is left by removal of the lysines. There was also a decrease in the mobility of probes on fibers exchanged with RLC-C31-MMTS on going from rigor to the SRX. Smaller decreases in mobility were also seen for fibers exchanged with RLC-C81-MMTS.

The changes in spectral intensity of the fluorescent probes discussed above do not provide a signal that would be useful for high throughput screens per se. Fortunately, the shift in the wavelength for the emission spectrum of MDCC attached to RLC-C5 provides a solution to this problem (Figs 7, 8 and 9). Because the fluorescence intensities at two wavelengths arise from excitation of one probe, the resulting ratio is independent of the amount of material being observed, excitation intensity and photo-bleaching. This provides the long sought for signal that can be used to search for potential therapeutic molecules that could increase resting muscle metabolism to treat obesity and Type 2 diabetes. Such screens are in progress and the results will be reported in the future.

## Supporting Information

S1 Fig Alignment of tarantula exoskeletal myosin RLC (Uniprot B4XT43, PDB 3DTP) with mouse fast muscle myosin RLC (Uniprot P97457). Output of the EMBOSS Needle Pairwise Sequence Alignment tool.
(TIF)

S2 Fig Bar graph of the Table 1 data. (TIF)

S3 Fig The structures of the probes employed in this study. The probes are: 4-maleimido-2,2,6,6,-tetramethyl-l-piperidinyloxy (MSL), 4-iodoacetamido-2,2,6,6,-tetramethyl-l-piperidinyloxy (IASL) (1-oxyl-2,2,5,5-tetramethylpyrroline-3-methyl)methanethiosulfonate (MMTS), 3-(bromomethyl)-2,5,6-trimethyl-1H,7H-pyrazolo[1,2-a]pyrazole-1,7-dione (BIMANE); 7-Diethylamino-3-[N-(2-maleimidoethyl)carbamoyl]coumarin (MDCC); 2-(4'-maleimidylanilino)naphthalene-6-sulfonic acid (MIANS).
(TIF)

S4 Fig EPR spectra of fibers exchanged with RLC-C81-MMTS. EPR spectra of fibers exchanged with RLC-C81-MMTS with the fiber axis perpendicular to the magnetic field. The fibers are in a relaxing solution, ATP plus bebbistatin, blue or in rigor, red. The greater Intensity at high and low magnetic field in the spectrum of the relaxed fibers indicates that the probes are more immobilized in relaxation and the SRX than in rigor. The center-field of the spectra is 0.3490 T and the sweep width is 10 mT.
(TIF)

S5 Fig EPR spectra of fibers exchanged with RLC-C5-MMTS. EPR spectra of fibers exchanged with RLC-C5-MMTS with the fiber axis perpendicular to the magnetic field. The fibers are in a relaxing solution, ATP plus bebbistatin, blue or in rigor, red. The greater Intensity at high and low magnetic field in the spectrum of the relaxed fibers indicates that the probes are more immobilized in relaxation and the SRX than in rigor. The center-field of the spectra is 0.3490 T and the sweep width is 10 mT.
(TIF)

S6 Fig Mouse skeletal muscle RLC sequence and chicken smooth muscle RLC alignment. Conserved residues (blue), non-conserved residues (green) and mutants (red) are highlighted as in Fig 3.
(TIF)
