# Supplementary figures and images for: Spectroscopic Studies of the Super Relaxed State of Skeletal Muscle
Source: PLoS One. 2016 Aug 1;11(8):e0160100. doi: 10.1371/journal.pone.0160100 (PMC4968846; doi:10.1371/journal.pone.0160100)

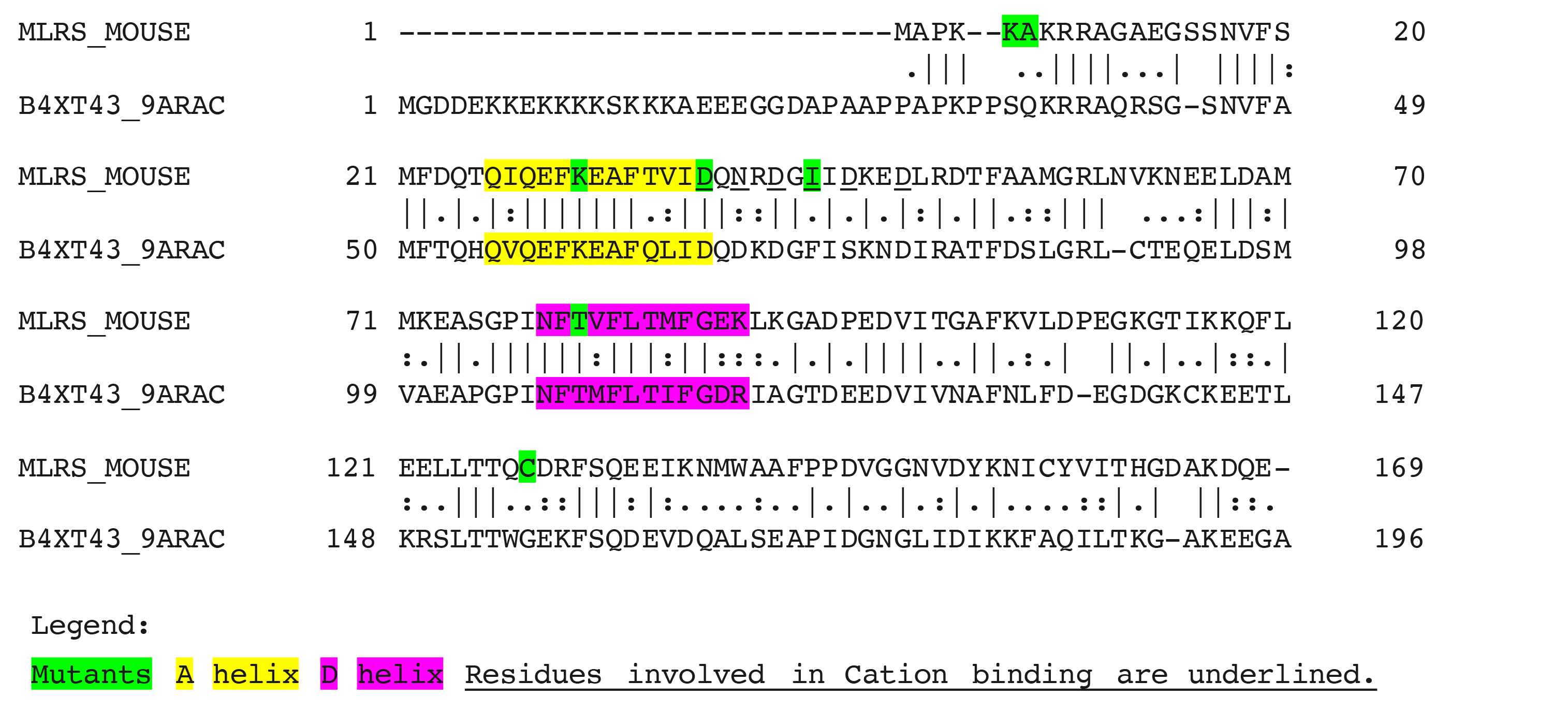

Supplement: S1 Fig — Output of the EMBOSS Needle Pairwise Sequence Alignment tool. (TIF) [file pone.0160100.s001.tif]

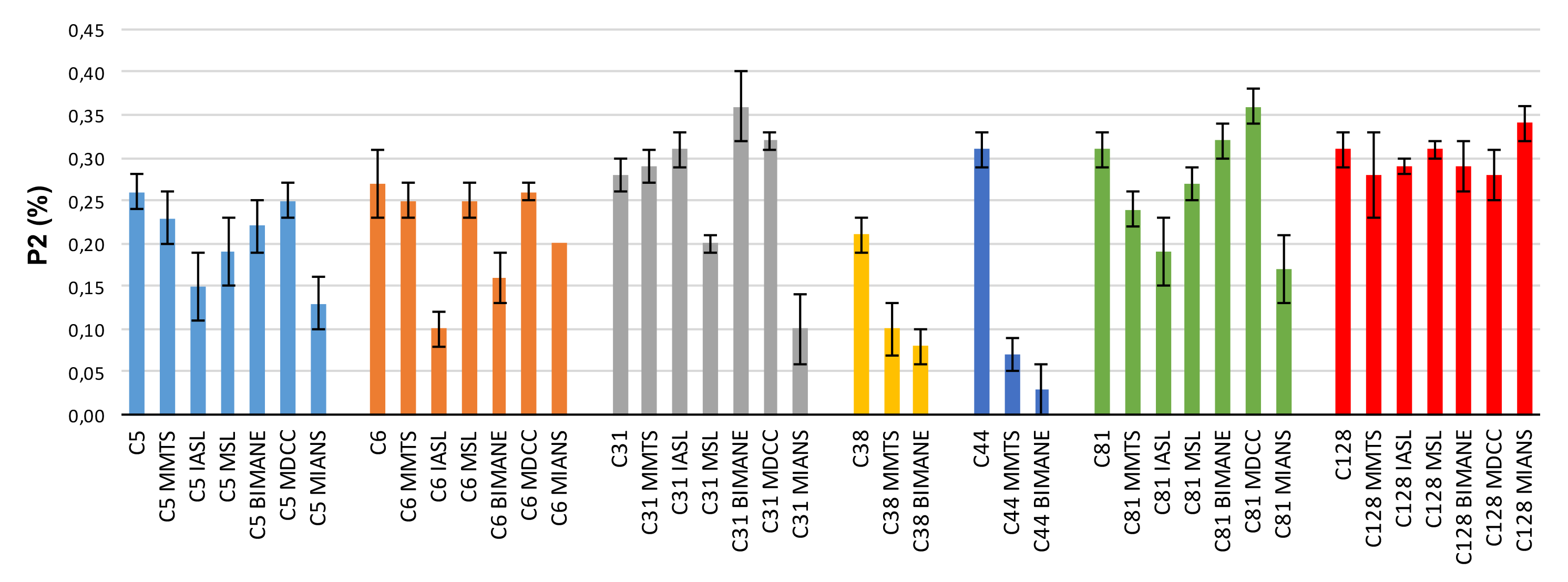

Supplement: S2 Fig — (TIF) [file pone.0160100.s002.tif]

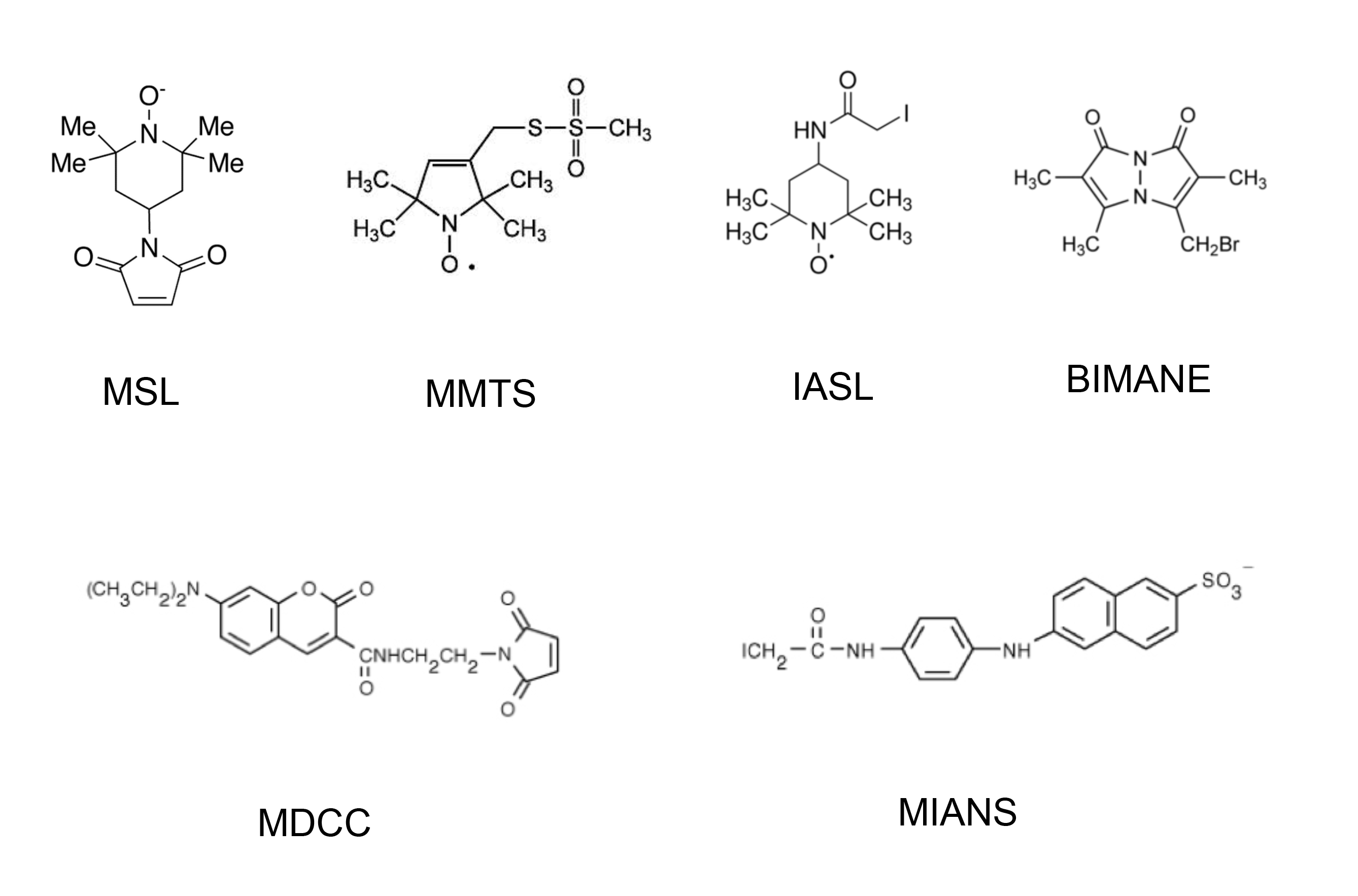

Supplement: S3 Fig — The probes are: 4-maleimido-2,2,6,6,-tetramethyl-l-piperidinyloxy (MSL), 4-iodoacetamido-2,2,6,6,-tetramethyl-l-piperidinyloxy (IASL) (1-oxyl-2,2,5,5-tetramethylpyrroline-3-methyl)methanethiosulfonate (MMTS), 3-(bromomethyl)-2,5,6-trimethyl-1H,7H-pyrazolo[1,2-a]pyrazole-1,7-dione (BIMANE); 7-Diethylamino-3-[N-(2-maleimidoethyl)carbamoyl]coumarin (MDCC); 2-(4'-maleimidylanilino)naphthalene-6-sulfonic acid (MIANS). (TIF) [file pone.0160100.s003.tif]

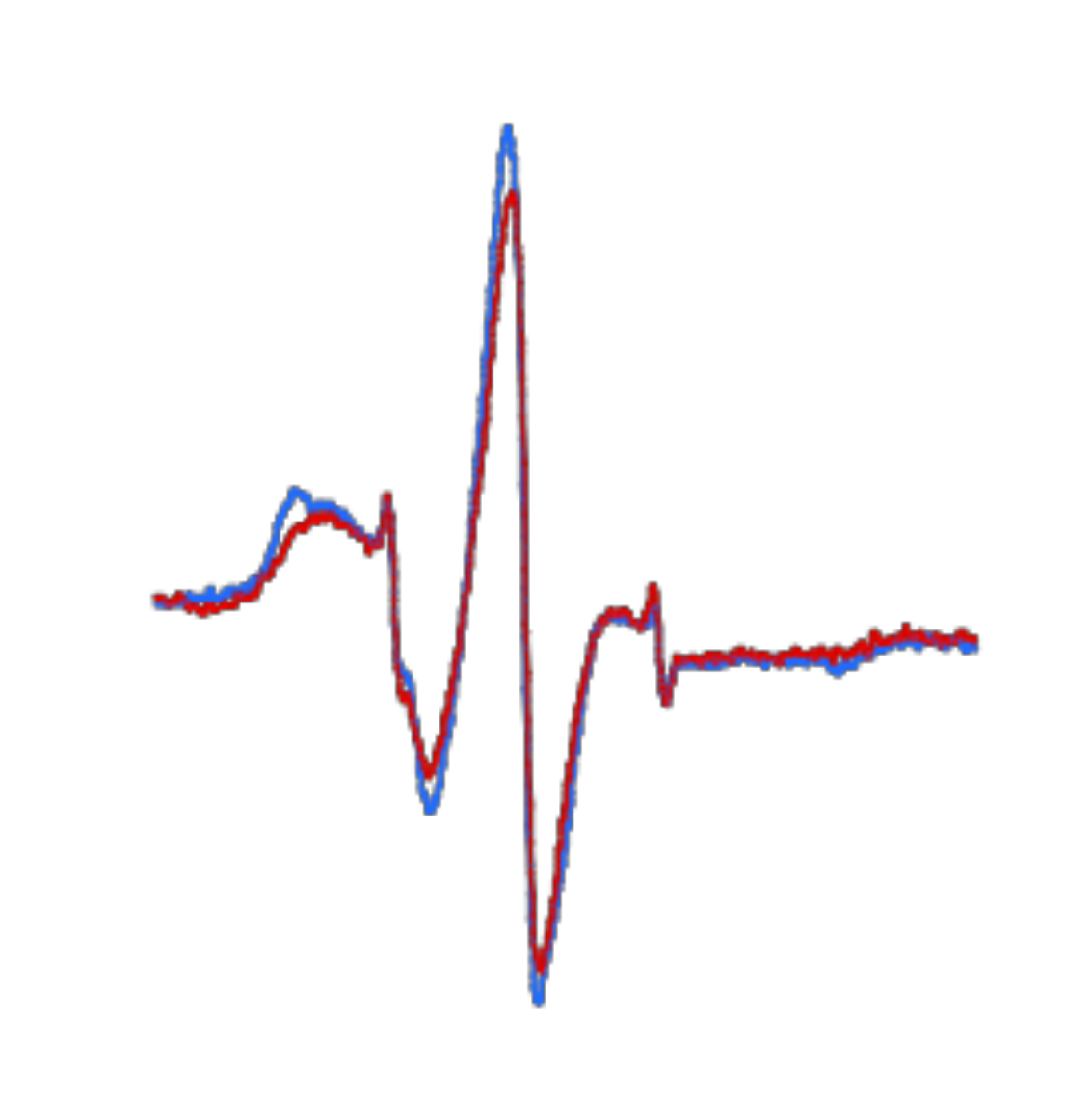

Supplement: S4 Fig — EPR spectra of fibers exchanged with RLC-C81-MMTS with the fiber axis perpendicular to the magnetic field. The fibers are in a relaxing solution, ATP plus bebbistatin, blue or in rigor, red. The greater Intensity at high and low magnetic field in the spectrum of the relaxed fibers indicates that the probes are more immobilized in relaxation and the SRX than in rigor. The center-field of the spectra is 0.3490 T and the sweep width is 10 mT. (TIF) [file pone.0160100.s004.tif]

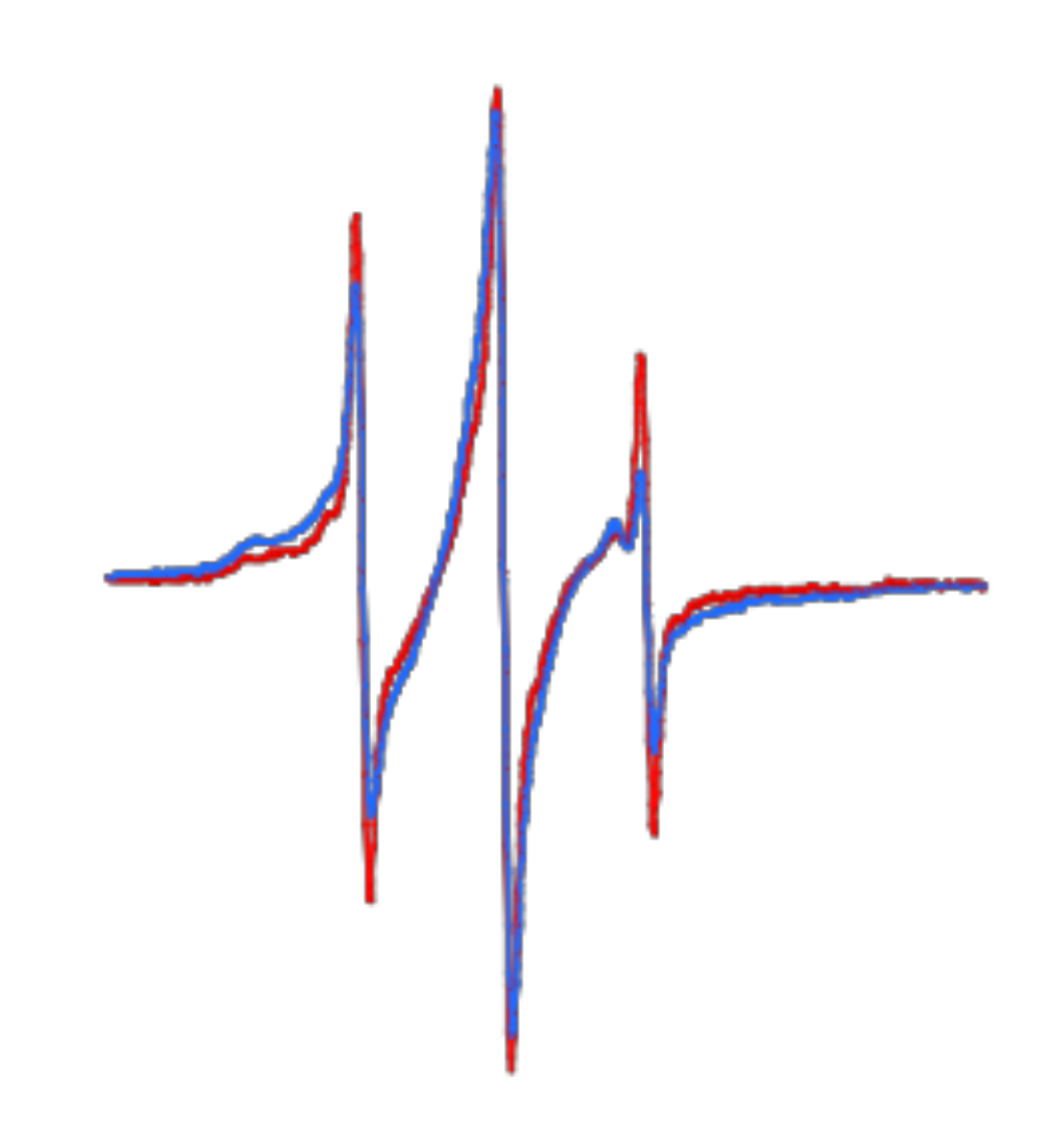

Supplement: S5 Fig — EPR spectra of fibers exchanged with RLC-C5-MMTS with the fiber axis perpendicular to the magnetic field. The fibers are in a relaxing solution, ATP plus bebbistatin, blue or in rigor, red. The greater Intensity at high and low magnetic field in the spectrum of the relaxed fibers indicates that the probes are more immobilized in relaxation and the SRX than in rigor. The center-field of the spectra is 0.3490 T and the sweep width is 10 mT. (TIF) [file pone.0160100.s005.tif]

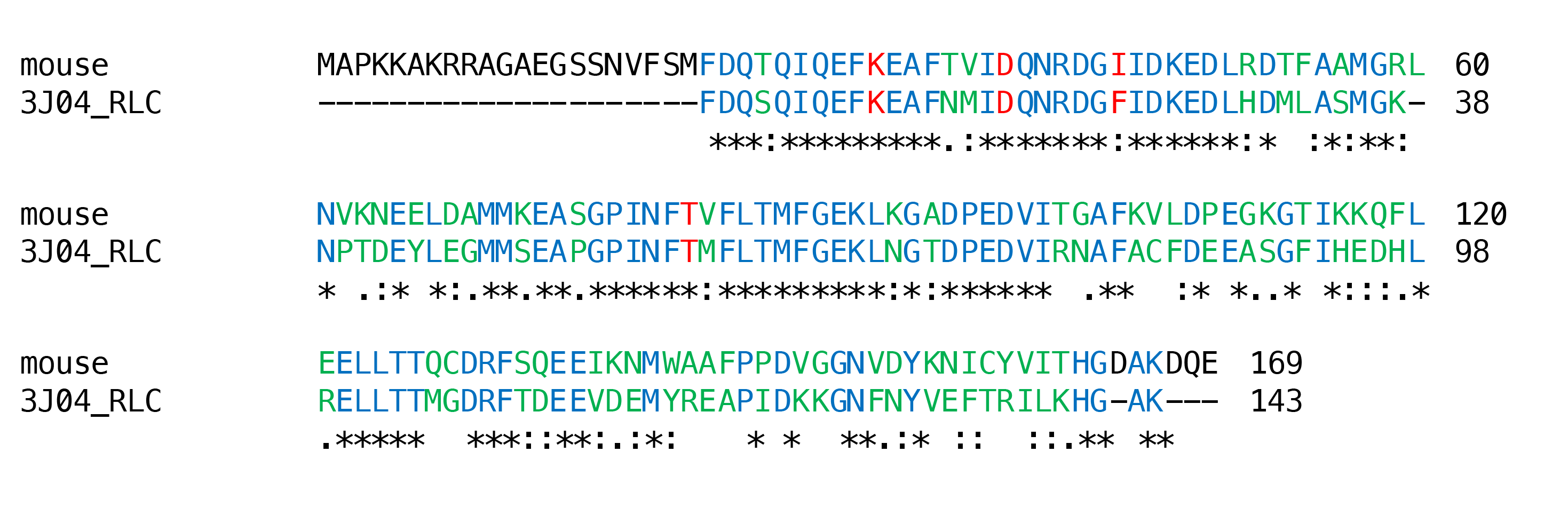

Supplement: S6 Fig — Conserved residues (blue), non-conserved residues (green) and mutants (red) are highlighted as in Fig 3. (TIF) [file pone.0160100.s006.tif]
